# Biochemical and molecular features of tetrahydrobiopterin deficiency in Fujian Province, southeastern China

**DOI:** 10.3389/fgene.2023.1250568

**Published:** 2023-08-11

**Authors:** Xiaolong Qiu, Peiran Zhao, Jinying Luo, Guilin Li, Lin Deng, Yinglin Zeng, Liangpu Xu, Jinfu Zhou

**Affiliations:** ^1^ Genetic Diagnosis and Therapy Center, Fujian Key Laboratory for Prenatal Diagnosis and Birth Defect, Fujian Maternity and Child Hospital, College of Clinical Medicine for Obstetrics & Gynecology and Pediatrics, Fujian Medical University, Fuzhou, China; ^2^ Obstetrics and Gynecology Department, Fujian Maternity and Child Hospital, College of Clinical Medicine for Obstetrics & Gynecology and Pediatrics, Fujian Medical University, Fuzhou, China; ^3^ Department of Preventive Medicine, School of Public Health, Fujian Medical University, Fuzhou, China

**Keywords:** tetrahydrobiopterin deficiency, newborn screening, 6-pyruvoyl-tetrahydropterin synthase, variant spectrum, southeastern China

## Abstract

The estimated prevalence of tetrahydrobiopterin deficiency (BH4D) and the mutational spectrum of the causal 6-pyruvoyl-tetrahydropterin synthase (*PTS*) gene vary widely according to race and region. This study assessed the prevalence and genetic characteristics of BH4D in Fujian Province, southeastern China. A total of 3,204,067 newborns were screened between 2012 and 2022 based on the phenylalanine level and the phenylalanine/tyrosine ratio in dried blood spots. Differential diagnosis was determined by the urine purine spectrum, dihydropteridine reductase activity in red blood cells, and genetic testing. The *PTS* mutation spectrum and genotypes were determined by next-generation sequencing. A total of 189 newborns were diagnosed with hyperphenylalaninemia (HPA) over the study period, including 159 with phenylalanine hydroxylase deficiency and 30 with BH4D. Therefore, the prevalence of BH4D in Fujian was 9.36 per 1,000,000 live births (30/3,204,067) and the proportion of BH4D among patients with HPA was 15.87% (30/189). A total of 58 *PTS* alleles were identified in the 29 patients with PTS deficiency (PTPSD), and those alleles were composed of 10 different variants, including eight missense variants and two splice-site variants. The most prevalent variants were c.155A>G, p.Asn52Ser (44.83%); c.259C>T, p.Pro87Ser (39.66%); and c.84-291A>G, p.Tyr27Argfs*8 (3.45%). The predominant genotype was c [155A>G]; [259C>T] (11/29, 37.93%). The prevalence of BH4D and the spectrum of associated *PTS* mutations were successfully determined for the first time in Fujian Province, southeastern China. Since the mutation spectrum of *PTS* is region-specific, such data will facilitate molecular diagnosis and genetic counseling in PTPSD cases.

## 1 Introduction

Hyperphenylalaninemia (HPA) is the most common autosomal recessive metabolic disorder, characterized by increased levels of amino acids caused by a deficiency in phenylalanine hydroxylase (PAH) or its cofactor tetrahydrobiopterin (BH4) (Çıkı et al., 2021). Differential diagnosis of PAH deficiency (PAHD) and BH4 deficiency (BH4D) can be achieved through the urine purine spectrum, determination of dihydropteridine reductase (DHPR) activity in red blood cells, and genetic testing (Lin et al., 2022; Vela-Amieva et al., 2022). In addition to increasing the blood phenylalanine (Phe) level, BH4D can decrease the synthesis of neurotransmitters since BH4 is also a significant cofactor for tyrosine hydroxylase and tryptophan hydroxylase, the rate-limiting enzymes in dopamine and serotonin synthesis, respectively. Without effective treatment, BH4D can lead to the development of intellectual and movement disorders. Since the treatment methods used for PAHD and BH4D differ, timely differential diagnosis is particularly crucial. BH4D can be caused by a deficiency in one of the five enzymes responsible for BH4 synthesis and recycling, including GTP cyclohydrolase I, 6-pyruvoyl-tetrahydropterin synthase (PTS), septerin reductase, pterin-4-carbinolamine dehydratase, and DHPR (Wang et al., 2018; Yuan et al., 2021). Among the enzymes, PTS deficiency (PTPSD) is the most common form of BH4D, which is caused by mutations in the *PTS* gene.

Newborn screening (NBS) programs for HPA have been implemented in many countries, resulting in significant improvements in BH4D early diagnosis and treatment (Shintaku and Ohwada, 2013; Khani et al., 2021). BH4D incidence is approximately 1%–3% among patients with HPA worldwide (Dhondt and Hayte, 2002; de Baulny et al., 2007; Dhondt, 2010; Wang et al., 2021). However, its incidence is higher in East Asia (Chiu et al., 2012). Based on a national cross-sectional survey of 107 million cases subject to NBS in mainland China from 2013 to 2019, the incidence of BH4D was estimated to be 3.8 per 1,000,000 live births, although the incidence rate varied greatly in different geographical locations (Yuan et al., 2021). Moreover, over 95% of PTPSD cases in mainland Chinese patients with BH4D are caused by *PTS* gene mutation (Ye et al., 2013; Han et al., 2015; Li et al., 2018). Although a single-center study on BH4D was conducted in Quanzhou (Lin et al., 2022) and Xianmen (Wang et al., 2019), Fujian Province, southeastern China, the overall prevalence and mutation spectrum of BH4D in Fujian have not been reported.

In the present study, the mutation prevalence and spectrum of *PTS* in patients diagnosed with BH4D were evaluated based on a multi-center and large-scale NBS program in Fujian Province, southeastern China. The biochemical features of patients with BH4D were also analyzed.

## 2 Methods

### 2.1 Study population

A total of 3,204,067 newborns (1,490,053 females and 1,714,014 males) screened across NBS centers in Fujian Province from January 2012 to December 2022 were included in the present study. The research protocol was approved by the Ethics Committee of Fujian Provincial Maternity and Child Hospital (permission no. 2017-037). Written informed consent was obtained from the guardians of all participants.

### 2.2 Newborn screening and biochemical analysis

The NBS workflow was based on a previously described procedure (Zhou et al., 2022). In brief, dried blood spot (DBS) samples were collected and transported via a cold-chain transportation system to the corresponding NBS center. The Phe concentration in the DBS samples was quantified using a DEFIA-1420 semi-auto time-resolved fluoroimmunoassay analyzer (Wallac, Finland) or an ACQUITY TQD tandem mass spectrometry (MS/MS) system (Waters, USA) with a Neonatal Phenylalanine kit (PerkinElmer, Turku, Finland). The cutoff value of Phe was set to 2.0 mg/dL (fluorometric method) or 120 μmol/L (MS/MS). When the Phe levels of the newborns exceeded the threshold, the families were contacted to undergo repeated testing using MS/MS. A consistent Phe level >120.0 μmol/L and Phe/tyrosine (Tyr) ratio >2.0 was used to establish a diagnosis of HPA.

### 2.3 Classification of HPA and differential diagnosis

Analysis of the urine purine spectrum, determination of DHPR activity in red blood cells, and genetic testing were performed to differentiate between PAHD and BH4D among newborns diagnosed with HPA. Biopterin (B) and neopterin (N) levels in the urine, and DHPR activity in DBS samples were measured simultaneously at the Xin Hua Hospital affiliated with Shanghai Jiao Tong University Medical School.

### 2.4 Urine purine spectrum analysis

Firstly, 100 mg ascorbic acid was added to 10 mL fresh urine to keep BH4 in its reduced form. After centrifugation, 50 µL of the supernatant was mixed with 450 µL of H_2_O. Then, 50 µL of 0.1 mol/L HCl and 10 mg MnO_2_ was added to the diluted urine and it was allowed to incubate for 15 min at room temperature. The mixture was centrifuged at 13,000 × *g* for 10 min. The supernatant was used for the measurement of B and N by high-performance liquid chromatography.

### 2.5 DHPR activity test

The DHPR activity was measured based on spectrophotometric monitoring of the formation of ferrocytochrome C in a coupled reaction, as describedin a previously published study (Arai et al., 1982). Quality assurance of the DHPR assay was confirmed by monitoring the dried blood spot elutes from a normal adult and a DHPR deficient patient.

### 2.6 Genotype analysis

Genomic DNA was isolated from DBS samples using a QIAamp DNA Mini Kit (Tiangen Biotech, China) according to the manufacturer’s instructions. Targeted next-generation sequencing was used to detect a target sequencing panel of 94 genes (including *PAH*, *PTS*, *GCH1*, *QDPR*, and *PCBD1*) related to inborn metabolic errors for the patients and their parents using the Biosan (Zhejiang, China) platform, as describedin a previous study (Zhou et al., 2022). Genetic variants were classified according to the American College of Medical Genetics and Genomics guidelines (https://clinicalgenome.org).

## 3 Results

### 3.1 Newborn screening for BH4D

**FIGURE 1:**
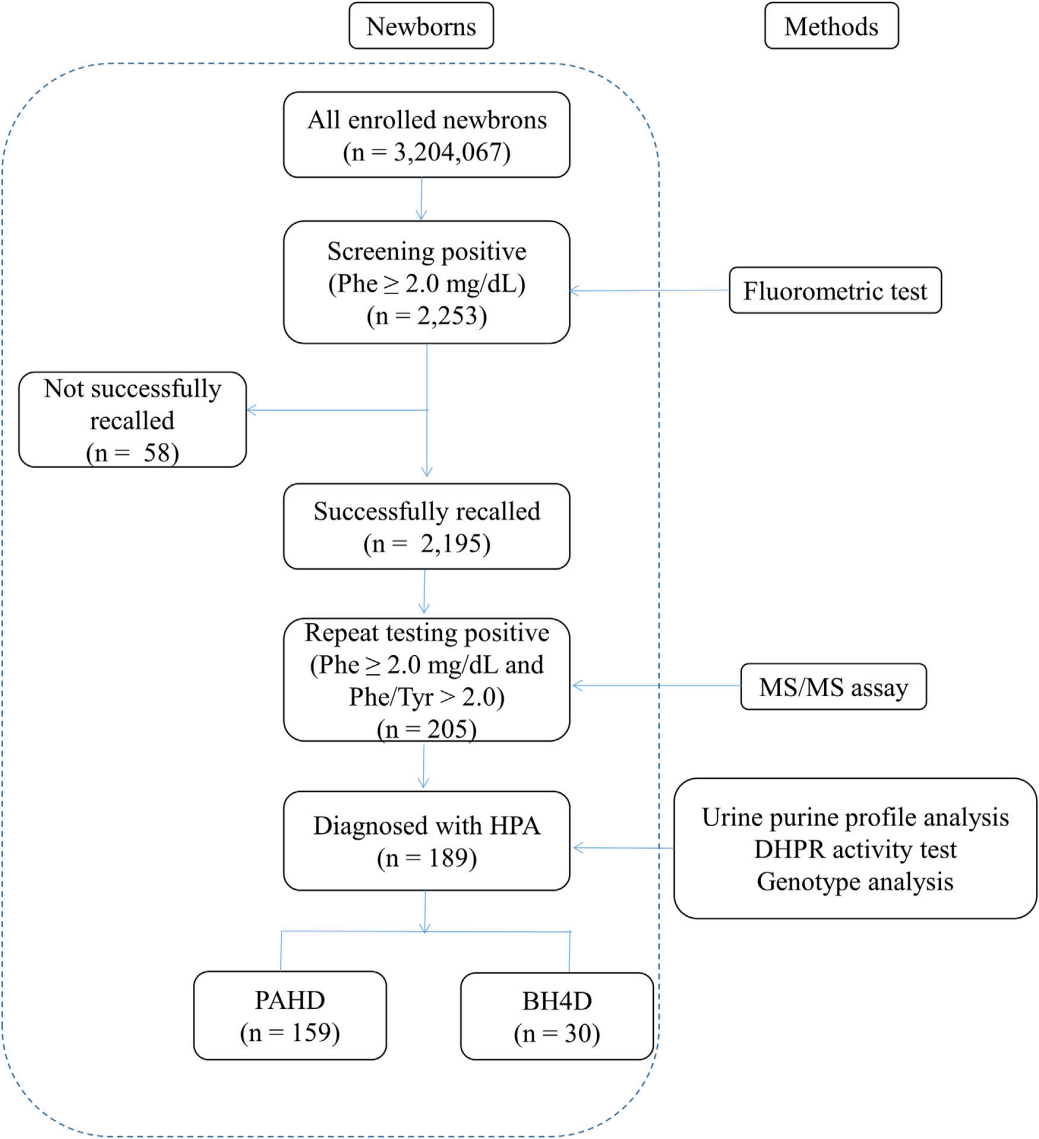
Workflow of screening of newborns for hyperphenylalaninemia. Phe, phenylalanine; Tyr, tyrosine; HPA, hyperphenylalaninemia; PAHD, phenylalanine hydroxylase deficiency; BH4D, tetrahydrobiopterin deficiency.

Among the 3,204,067 newborns screened over 11 years, 2,253 had elevated concentrations of Phe at the initial NBS, yielding a positivity rate of 0.07%. Subsequently, 2,195 newborns with elevated Phe levels were successfully recalled, and 205 newborns with positive results confirmed using MS/MS were subjected to analysis of the urine purine spectrum, determination of DHPR activity, and genetic testing. Finally, 189 newborns were diagnosed with HPA, with a positive predictive value of 8.61% (189/2195) (Figure 1). Therefore, the estimated HPA incidence in Fujian Province is 1 in 16,952 newborns. Among the patients with HPA, 159 had PAHD and 30 had BH4D. Therefore, the prevalence of BH4D in Fujian Province was 9.36 per 1,000,000 live births (30/3,204,067) and the ratio of BH4D among patients with HPA was 15.87% (30/189).

### 3.2 Biochemical characteristics of patients with BH4D

The 30 patients with BH4D, including 29 with PTPSD (96.67%) and one with DHPR deficiency (3.33%), were further investigated. The mean Phe level and Phe/Tyr ratio among patients with PTPSD were 898.5 ± 454.3 μmol/L and 12.91 ± 7.62, respectively. All 25 patients with PTPSD who underwent urine purine spectrum and DHPR activity analyses had low or nearly no B, with an overall very low percentage of B [B/(B + N)%, B%] (<5%), and normal DHPR activity (Table 1). The patient with DHPR deficiency had a normal B concentration (2.89 mmol/molCr) and B% (45.64%), but very low DHPR activity (5.5% that of the control).

**TABLE 1:** Biochemical and genetic features of 29 PTPSD patients.

Patient no.	Gender	Phe (mg/dL)	MS/MS		Age at time of diagnosis	B (mmol/molCr)	N (mmol/molCr)	B% [B/(N + B)%]	DHPR (%)	Genotype	
			Phe (µmol/L)	Phe/Tyr						Maternal allele	Paternal allele
‘1	F	12.47	971	12.86	1 m	0.06	7.70	0.77	97.45	c.155A>G	c.155A>G
2	F	10.93	517.4	6.78	35 d	n.d.	n.d.	n.d.	n.d.	c.155A>G	c.259C>T
3	F	7.85	960	7.98	28 d	0.09	9.46	0.94	86.5	c.73C>G	c.259C>T
4	F	4.09	1070.4	12.12	32 d	0.16	41.4	0.38	84.48	c.155A>G	c.259C>T
5	F	17.8	1400.01	22.15	36 d	0.08	1.21	6.2	102	c.155A>G	c.272C>T
6	F	8.87	543	7.76	42 d	n.d.	n.d.	n.d.	n.d.	c.155A>G	c.155A>G
7	F	13.25	870	15.34	33 d	0.35	19.7	1.75	96.8	c.166G>A	c.317C>T
8	M	9.2	785.5	12.5	24 d	0.37	8.89	4.0	92.50	c.84-291A>G	c.155A>G
9	M	7.345	1161	21.33	2 m	0.17	7.48	2.22	85.73	c.259C>T	c.155A>G
10	M	14.68	874.8	14.58	25 d	0.23	9.49	2.37	98.47	c.155A>G	c.259C>T
11	M	9.41	682.8	8.74	34 d	0.08	5.67	1.39	79.85	c.155A>G	c.259C>T
12	F	31.05	1944.66	28	1 m	0.04	10.62	0.37	103.2	c.84-291A>G	c.259C>T
13	F	5	557.87	5.47	27 d	0.11	5.39	2.0	66.85	c.259C>T	c.259C>T
14	F	24.6	1479	21.23	23 d	0.07	6.2	1.11	93.50	c.155A>G	c.259C>T
15	M	7.384	324.56	5.87	22 d	0.21	4.21	4.75	88.73	c.286G>A	c.155A>G
16	F	9.2	531.84	7.13	1 m	0.31	31.4	0.98	94.50	c.155A>G	c.259C>T
17	F	7.52	393	5.89	35 d	0.03	6.78	0.44	79.85	c.155A>G	c.155A>G
18	M	15.90	958.2	13.3	27 d	n.d.	n.d.	n.d.	n.d.	c.155A>G	c.259C>T
19	F	13.5	517.51	6.34	28 d	n.d.	n.d.	n.d.	n.d.	c.259C>T	c.259C>T
20	F	13.06	1308.6	20.23	21 d	0.05	3.07	1.6	97.65	c.155A>G	c.259C>T
21	F	7.97	670.35	7.76	29 d	0.47	23.1	1.99	68.5	c.244-2A>T	c.259C>T
22	M	11.23	614.56	9.105	36 d	0.02	65	0.03	94.35	c.155A>G	c.259C>T
23	F	8.01	1049.1	7.89	25 d	0.75	33.17	2.21	89.5	c.259C>T	c.259C>T
24	F	10.34	2356.35	38.58	22 d	0.69	96.3	0.71	64.48	c.155A>G	c.155A>G
25	F	6.62	995.63	19.45	36 d	0.08	66.22	0.12	100	c.155A>G	c.155A>G
26	M	7.45	770.35	8.76	26 d	0.18	10.91	1.09	88.45	c.155A>G	c.155A>G
27	M	5.3	319.31	5.81	22 d	0.1	19.36	0.51	79.25	c.259C>T	c.259C>T
28	M	10.73	695.62	11.35	23 d	n.d.	n.d.	n.d.	n.d.	c.259C>T	c.170T>A
29	F	10.61	733.8	10.25	34 d	0.13	18.18	0.7	95.75	c.155A>G	c.259C>T

The reference range of Phe and Phe/Tyr using MS/MS, was 20–105 μmol/L and 0.16–1.50, respectively.

The reference range of B, N, and B% were 1.73–3.68 mmol/mol Cr, 0.90–7.49 mmol/mol Cr, and 26.2%–68.4%, respectively.

Abbreviation: M, male, F, female, d, day, m, month, N, neopterin, B, biopterin, n.d., not determined, PTPSD, 6-pyruvoyl-tetrahydropterin synthase deficiency.

### 3.3 Variant spectrum of the *PTS* gene

**FIGURE 2:**
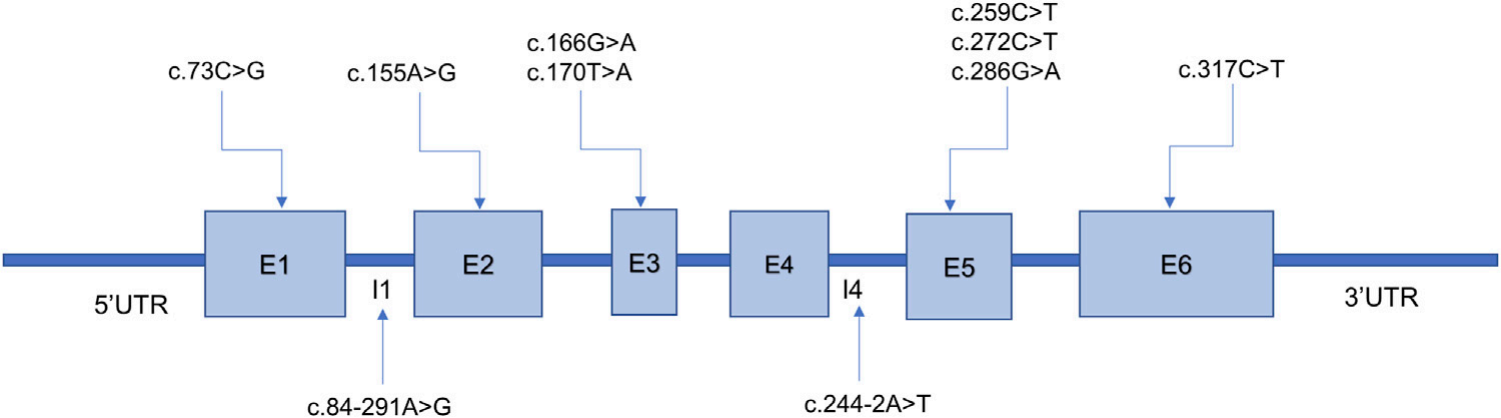
Ten variants identified in the 6-pyruvoyl-tetrahydropterin synthase (*PTS*) gene among patients with PTS deficiency from Fujian Province. E, exon; I, intron. The *PTS* transcript version access number is NM_000317.3.

A total of 58 *PTS* alleles were identified in the 29 patients with PTPSD, and those alleles were composed of 10 different variants, including eight (80%) missense variants and two (20%) splice-site variants (Table 2). At the amino acid sequence level, the three most prevalent variants accounted for 87.93% of the total: c.155A>G, p.Asn52Ser (44.83%); c.259C>T, p.Pro87Ser (39.66%); and c.84-291A>G, p.Tyr27Argfs*8 (3.45%). Only one novel variant, c.170T>A, p.Val57Glu in the *PTS* gene was identified in this study, the other variants observed in this study have been reported before. Excluding exon 4, the variants were relatively evenly distributed along the *PTS* gene (Figure 2).

**TABLE 2:** *PTS* gene mutations in the PTPSD patients from Fujian province.

Pathogenic variants	Protein effect or trivial name	Gene region	Type	Pathogenicity classification	No. Of alleles	RF (%)	References
c.155A>G	p.Asn52Ser	Exon 2	Missense	P	26	44.83	Liu and Hsiao (1996)
c.259C>T	p.Pro87Ser	Exon 5	Missense	P	23	39.66	Liu and Hsiao (1996)
c.84-291A>G	p.Tyr27Argfs*8	Intron 1	Splice	P	2	3.45	Gu et al. (2009)
c.73C>G	p.Arg25Gly	Exon 1	Missense	LP	1	1.72	Liu et al. (1998)
c.166G>A	p.Val56Met	Exon 3	Missense	P/LP	1	1.72	Thöny and Blau (1997)
c.170T>A	p.Val57Glu	Exon 3	Missense	VUS	1	1.72	This study
c.244-2A>T	p. (?)	Intron 4	Splice	LP	1	1.72	Wang et al. (2018)
c.272C>T	p.Lys91Arg	Exon 5	Missense	P	1	1.72	Ye et al. (2007)
c.286G>A	p.Asp96Asn	Exon 5	Missense	P/LP	1	1.72	Imamura et al. (1999)
c.317C>T	p.Thr106Met	Exon 6	Missense	P/LP	1	1.72	Thöny and Blau (1997)

Abbreviation: LP, likely pathogenic; P, pathogenic; VUS, variants of uncertain significance; RF, relative frequency; *PTS*, 6-pyruvoyl-tetrahydropterin synthase; PTPSD, 6-pyruvoyl-tetrahydropterin synthase deficiency. The number of *PTS*, transcription version is NM_000317.3.

### 3.4 Genotype distribution of patients with BH4D

All patients with PTPSD carried biallelic variants and were either compound heterozygous (n = 19) or homozygous (n = 10), with one variant originating from the mother and the other one originating from the father (Table 1). At the amino acid level, 11 distinct combinations were found in the 29 patients, and the most abundant genotypes observed were c.[155A>G]; [259C>T] (11/29, 37.93%), c.[155A>G]; [155A>G] (6/29, 20.69%), and c.[259C>T]; [259C>T] (4/29, 13.79%), as shown in Table 1. The patient with DHPR deficiency had a compound heterozygous mutation in the *DHPR* gene, and the genotype was c.[508G>C][523_525GCA>AGA].

## 4 Discussion

Early detection of HPA through NBS is crucial for timely intervention and prevention of adverse clinical symptoms in affected individuals. With the widespread application of the urine purine spectrum, DHPR activity in red blood cells, and next-generation sequencing, PAHD and BH4D can now be identified in a timely manner.

Only a few studies to date have investigated the prevalence of BH4D, with a global incidence of approximately 1 in 500,000 live births (Blau et al., 2018); however, wide variation in the estimated prevalence has been reported depending on the geographical location and ethnic composition of the different provinces in China (Wang et al., 2021; Xie et al., 2022). The prevalence of BH4D in the northern regions (4.1 per 1,000,000) of China is reportedly higher than that in the southern regions (1.6 per 1,000,000) (Yuan et al., 2021). The prevalence is up to 12.5 per 1,000,000 live births in Jiangxi Province, South China (Xie et al., 2022), and estimated at 15.5 and 9.44 per 1,000,000 live births in Quanzhou city (Lin et al., 2022) and Xianmen city (Wang et al., 2019) of Fujian Province, respectively. In the present study, 30 patients with BH4D were identified among 3.2 million NBS results, representing an incidence of 9.36 per 1,000,000 live births in Fujian Province. The incidence was much higher than the national average in China and is similar to that reported in Jiangxi Province, South China. The proportion of BH4D in HPA cases varies greatly in different regions of the world, with an incidence rate of 0.5% in Russia (Gundorova et al., 2021) and an incidence rate as high as 30% in Jordan (Carducci et al., 2020). A previous study estimated the proportion of BH4D among patients with HPA in mainland China to be 8.55% (Ye et al., 2009). However, significant regional differences in the proportion have also been observed, with a higher proportion in the southern region (Xie et al., 2022) and a lower proportion in the northern region (Han et al., 2015). In the present study, BH4D accounted for 15.87% of patients with HPA, which is similar to the proportion reported for Taiwan (Niu et al., 2010). In addition, 29 out of the 30 (96.67%) patients with BH4D in our study had PTPSD, which is consistent with previous studies (Ye et al., 2013; Han et al., 2015; Xie et al., 2022).

The human *PTS* gene is located on chromosome 11q22.3 and contains six exons encoding more than 500 nucleotides. More than 700 confirmed pathogenic *PTS* variants have been detected (http://www.hgmd.cf.ac.uk; data collected on 17 July 2023). The high-frequency regions of variants and the mutational spectrum of *PTS* vary widely by race and region. A previous study showed that exon 6 was the most frequently mutated region in Italy (Manti et al., 2020), whereas another study indicated that exon 5 was the most commonly mutated region in Chinese patients (Li et al., 2022). However, excluding exon 4, the variants were found to be relatively evenly distributed along the *PTS* gene in the newborns screened in the present study. The c.238A>G (p. Met80Val) mutation is highly prevalent in the Arab population, accounting for 33% (Almannai et al., 2019), whereas c.317C>T (p.Thr106Met) is the most common variant in Russia (Gundorova et al., 2021). In East Asian populations, the three most prevalent variants accounted for 87.93% of all variants: c.259C>T (p.Pro87Ser) (39.66%), c.155A>G (p.Asn52Ser) (15.62%), and c.286G>A (p.Asp96Asn) (10.51%) (Chiu et al., 2012). Some studies have reported that c.259C>T is the most prevalent variant in mainland China (Ye et al., 2013; Han et al., 2015; Xie et al., 2022). Ten distinct variants were detected in the present study, more than two-thirds of which were identified only once, revealing a high degree of genetic heterogeneity among patients with PTPSD in Fujian Province. The following three variants accounted for 87.93% of the total variants among the patients with PTPSD identified in our study: c.155A>G (p.Asn52Ser) (44.83%), c.259C>T (p.Pro87Ser) (39.66%), and c.84-291A>G (p.Tyr27Argfs*8) (3.45%), which is consistent with the mutation spectrum identified in Taiwan (Chiu et al., 2012). In addition, one novel variant, c.170T>A, p.Val57Glu in the *PTS* gene was identified in this study, which enriched the human genetic variation database.

The most prevalent genotypes among the patients with BH4D identified in the present study were c.[155A>G]; [259C>T] (11/29, 37.93%), c.[155A>G]; [155A>G] (6/29, 20.69%), and c.[259C>T]; [259C>T] (4/29, 13.79%), which is inconsistent with previous findings in other provinces in China. The most prevalent genotypes of patients with BH4D in Beijing and Shandong were c.[ 84-291A>G]; [286G>A] (4/11, 36.36%) and c.[259C>T]; [259C>T] (3/11, 27.27%), respectively. The results further highlight the regional and ethnic heterogeneity of variants contributing to BH4D in China.

All PTPSD patients received treatment with BH4, L-dopa, and 5-HTP. The median minimal starting dose of BH4 was 1 mg/kg/day, and adjusted according to Phe concentrations to achieve the target Phe concentration for the corresponding ageof the patient (Yang et al., 2014). When the Phe concentration of the patients with BH4 treatment is not well controlled, diet therapy will be used. The median minimal starting doses of L-dopa and 5-HTP were also 1 mg/kg/day, with increments of 1 mg/kg every 5–7 days as required to achieve target concentrations for the corresponding age and adjusted according to clinical symptoms and blood prolactin concentrations. In addition, the clinical outcomes of some patients, including physical development status and intelligence, are being evaluated and collected. We hope to gather data on and show the clinical outcomes for all patients and conduct an analysis of gene-phenotype correlation in the future.

## 5 Conclusion

The biochemical and molecular features of 30 non-related patients with BH4D were investigated. The estimated incidence of BH4D in Fujian Province is 9.36 per 1,000,000 live births. The prevalence of PTPSD among patients with BH4D was 96.67%. We successfully established the *PTS* mutation spectrum in Fujian Province for the first time and found that the c.155A>G variant was the most prevalent *PTS* variant. The most dominant genotype was c.[155A>G]; [259C>T] (37.93%). The mutation spectrum is region-specific and therefore should facilitate the improvement of the molecular diagnosis and genetic counseling of families affected by PTPSD.

## Data Availability

The original contributions presented in the study are included in the article/Supplementary material, further inquiries can be directed to the corresponding authors.
